# High-Entropy Double Perovskites with Tailored Multichannel Luminescence

**DOI:** 10.1021/jacs.5c18097

**Published:** 2025-12-11

**Authors:** Jie Xue, Jun Luo, Kin Ting Chang, Zihang Sun, Zonglong Zhu, Lingling Mao, Haipeng Lu

**Affiliations:** 1 Department of Chemistry, 58207 The Hong Kong University of Science and Technology, Clear Water Bay, Kowloon, Hong Kong 999077, China (SAR); 2 Department of Chemistry, 255310 Southern University of Science and Technology, Shenzhen, Guangdong 518055, China; 3 Department of Chemistry, 53025 City University of Hong Kong, Kowloon, Hong Kong 999077, China (SAR)

## Abstract

Entropy engineering has emerged as a versatile strategy for designing metastable materials with synergistic properties and functionalities. Here, we present a facile solution method that yields a new series of high-entropy metal-halide double perovskites (HE-DPs). Single crystals of HE-DPs with a general formula of Cs_2_M^I^M^III^Cl_6_ (M^I^ = Ag^+^, Na^+^; M^III^ = In^3+^, Sb^3+^, Ho^3+^, Er^3^^+^, Bi^3+^, Yb^3^^+^, Dy^3^^+^, or Tb^3^^+^) are obtained under mild conditions. Structural and elemental analyses demonstrate the formation of high-entropy single-phase single crystals with five elements occupying the trivalent M^III^ site. The incorporation of multiple trivalent metal ions in a high-entropy manner appears to drastically improve the ambient stability of double perovskites up to more than three months. The optical bandgap is found to decrease upon alloying at the M^III^ site. Additionally, the random distribution of lanthanide ions within the crystal structure results in synergic electronic interactions between the lanthanide host and lanthanide-lanthanide ions. The interaction between lanthanides and host induces both broadband emissions and sharp Ln^3+^ *f*–*f* transitions, while the lanthanide-lanthanide proximity leads to efficient NIR-to-visible photon upconversion. Our work underscores the high-entropy strategy for developing robust lanthanide perovskites featuring tailored, multichannel optical properties for advanced lighting, display, and sensing applications.

## Graphical abstract

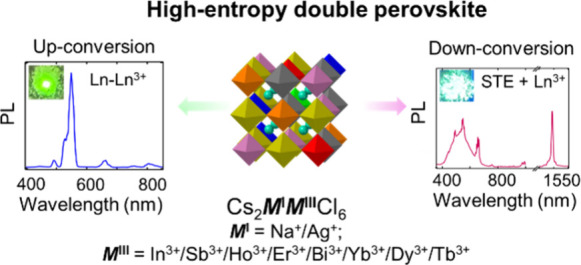

## Introduction

Halide perovskites have emerged as promising semiconductor materials, driving significant advancements in a wide range of optoelectronic applications including solar cells, light-emitting diodes, and photodetectors.^1–5^ The key to their success is the remarkable defect tolerance and excellent photophysical properties including high absorption coefficients, tunable bandgaps, and long carrier lifetimes.^6,7^ However, the widespread deployment of lead-based perovskites is hampered by the intrinsic toxicity of lead and their often-limited operational stability under ambient conditions.^8^ Lead-free double perovskites with a general formula of Cs_2_M^I^M^III^X_6_ (B^I^ = Ag^+^, Na^+^, Cu^+^, B^III^ = In^3+^, Sb^3+^, Bi^3+^, Fe^3+^, Ln^3+^, X = Cl^–^, Br^–^, I^–^) have garnered considerable attention as environmentally benign alternatives.^9,10^ Among these, lanthanide (Ln^3+^) based double perovskites are particularly attractive because of the unique optical and magnetic properties of Ln^3+^ ions.^11–17^ Despite the extensive chemical space predicted for lanthanide-based double perovskites, experimental synthesis of these compounds remains limited to date.^18,19^ This scarcity stems from a thermodynamic competition between the double perovskite phase (A_2_M^I^M^I^ ^I^ ^I^X_6_) and other stable phases, such as binary or ternary metal halides, and the severe hygroscopicity associated with lanthanide ions.^20,21^

To address these limitations, we propose an entropy engineering strategy for lanthanide-halide double perovskites. High-entropy (HE) engineering involves creating solid solutions with five or more principal elements in near-equimolar ratios (typically 5–35% each).^22–26^ This approach leads to high configurational entropy (Δ*S*_config_ > 1.5*R*, *R* is the gas constant) and presents a new paradigm for material discovery. By leveraging the stabilizing effect of high configurational entropy, the HE strategy can potentially overcome conventional solubility limits, promote the formation of single-phase solid solutions even with chemically disparate elements, and unlock synergistic “cocktail effects” arising from complex multi-ion interactions.^27–31^ This approach has proven successful in enhancing stability and tunability and achieving novel functionalities across various metals, oxides, and other material classes.^32–36^ However, conventional HE synthesis requires high-temperature synthesis procedures exceeding 1000 °C and complex processing techniques.^37–39^ Recently, Yang’s group showed a mild chemistry route toward HE semiconductors featuring the vacancy-ordered double perovskite structure.^40^ Song et al. synthesized a series of HE two-dimensional perovskites exhibiting enhanced air and humidity stability.^41^ These works suggest a unique opportunity for entropy engineering in stabilizing ionic semiconductors under mild reaction conditions.

Herein, we report the synthesis of a new series of high-entropy double perovskites (HE-DPs) with a general formula of Cs_2_M^I^M^III^Cl_6_ (M^I^ = Ag^+^, Na^+^; M^III^ = In^3+^, Sb^3+^, Bi^3+^, Er^3^^+^, Yb^3^^+^, Dy^3^^+^, Tb^3^^+^, or Ho^3+^). This was achieved by incorporating five elements at the trivalent M^III^ site, and one to two elements at the monovalent M^I^ site. Structural and elemental analyses suggest the formation of single-phase single crystals of HE-DPs. The entropy factor was shown to be critical for the stability of lanthanide double perovskites, where a positive correlation between the configurational entropy and air stability was observed. In general, high-entropy double perovskites display a higher air stability over those of medium- and low-entropy double perovskites. HE alloying also leads to a decreased optical bandgap in double perovskites. More interestingly, the random alloying of lanthanide ions leads to both broadband emissions arising from self-trapped exciton (STE) and sharp Ln^3+^ *f*–*f* transitions. On the other hand, the tailored lanthanide-lanthanide interaction in HE-DP further gives rise to efficient NIR-to-visible photon upconversion. This work establishes the HE strategy as a powerful tool for designing robust, multifunctional halide perovskite semiconductors.

## Results and Discussion

### Synthesis and Crystal Structures

A typical double perovskite structure is represented by the formula of A_2_M^I^M^III^X_6_. Here, we employed a hydrothermal method to synthesize single crystals of HE-DPs alloyed at M^I^- and M^III^-sites, while keeping the A site and halide fixed, namely, Cs_2_M^I^M^III^Cl_6_. The M^I^ site is alloyed with Na^+^ and Ag^+^, while the M^III^ site is alloyed with In^3+^, Sb^3+^, Bi^3+^, and various Ln^3+^ ions (Er^3^^+^, Yb^3^^+^, Dy^3^^+^, Tb^3^^+^, or Ho^3+^). A randomly mixed solid solution of Cs_2_{M^I^M^III^}­Cl_6_ will have a high atomic configurational entropy (Δ*S*_config_), given for ideal solutions by^42^

$$\Delta S_{\mathrm{config}}=-R\left(\sum_{i=1}^{N}x_{i}\ln\;x_{i}+\sum_{j=1}^{P}y_{j}\ln\;y_{j}\right)$$

where *N* and *P* represent the number of ions on M^I^ and M^III^ sites of double-perovskite, respectively, and *x*_*i*_ and *y*_*j*_ are the mole fractions of ions *i* ^th^ and *j* ^th^, respectively. HE materials are generally referred to those systems with Δ*S*_config_ larger than 1.5*R*.^42^ We envision that the double perovskite structure (Figure 1a) with two distinct octahedra centers can potentially provide an even larger chemical space for entropy engineering, when compared to simple perovskite or vacancy-ordered double perovskite structures. For instance, if the M^I^ site is fixed to one element (*N* = 1, *x*_*i*_ = 1), HE-DP can be obtained by alloying the M^III^ sites with five or more elements. If the M^I^ site is also alloyed (*N* = 2), HE-DP can be readily obtained by alloying three or more elements at the M^III^ sites.

**Figure 1:**
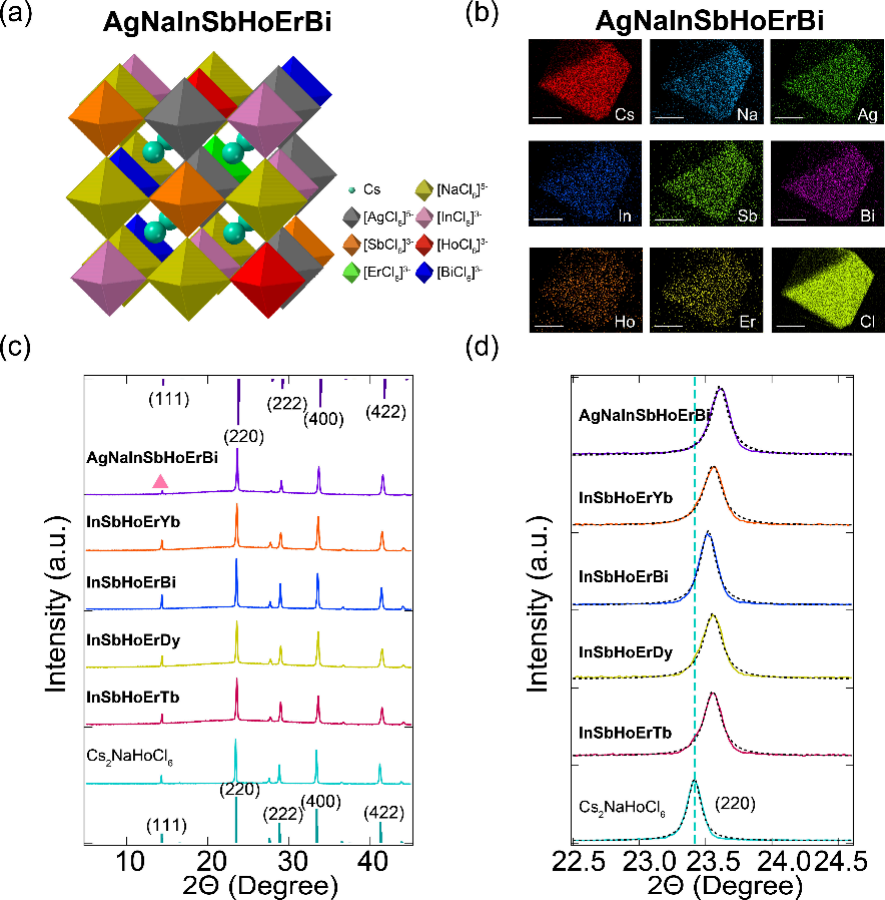
(a) The crystal structure of Cs_2_{NaAg}_1_{InSbHoErBi}_1_Cl_6_ (AgNaInSbHoErBi). (b) SEM-EDX results for the same HE-DP crystal. The scale bar is 10 μm in the SEM images. (c) P-XRD patterns of the synthesized double perovskites compared with the standard reference patterns of Cs_2_NaHoCl_6_ (bottom) and (AgNaInSbHoErBi) (up). (d) Magnified P-XRD patterns of the (220) peaks from Cs_2_NaHoCl_6_ and high-entropy double perovskites. The black-dashed lines illustrate the single Lorentzian fit.

As such, we first focus on alloying five trivalent metal ions including In^3+^, Sb^3+^, Bi^3+^, and various Ln^3+^ ions at the M^III^ site while keeping the M^I^ site of Na^+^ unchanged. Following a comprehensive optimization of the reaction conditions, we successfully obtained HE-DP single crystals with five M^III^ elements, Cs_2_Na­{InSbHoErTb}­Cl_6_ (**InSbHoErTb**), Cs_2_Na­{InSbHoErDy}­Cl_6_ (**InSbHoErDy**), Cs_2_Na­{InSbHoErBi}­Cl_6_ (**InSbHoErBi**), and Cs_2_Na­{InSbHoErYb}­Cl_6_ (**InSbHoErYb**). We then explored HE-DP by simultaneously alloying Na^+^ and Ag^+^ ions at the M^I^ site, giving a total of seven elements at the octahedra sites, namely, Cs_2_{NaAg}­{InSbHoErBi}­Cl_6_ (**AgNaInSbHoErBi**). Elemental analysis based on inductively coupled plasma optical emission spectrometry (ICP-OES) shows that the concentration of each alloying element is in the range of 5–35%, suggesting them as high-entropy systems (Table S1).^23^ The calculated atomic configurational entropy Δ*S*_config_ values for the as-synthesized double perovskites are summarized in Table S1, where all compounds show Δ*S*_config_ values larger than 1.5*R*.

To further confirm the structure, composition, and phase purity, additional structural and elemental analyses were conducted. The crystal structure and composition of a representative HE-DP single crystal (**AgNaInSbHoErBi**) were first determined using single-crystal X-ray diffraction (SC-XRD) (Figure 1a). Crystallographic details and refinement parameters are shown in Tables S2–S4. SC-XRD analysis yields a rock-salt-ordered double perovskite structure featuring alternating [M^I^Cl_6_] and [M^III^Cl_6_] octahedra that share corners. Energy-dispersive X-ray spectroscopy (EDX) mapping (Figure 1b) indicates the incorporation of all seven elements homogeneously distributed within a single crystal domain. EDX analysis of other ground HE-DPs yields similar results (Figures S1–S4), confirming the homogeneous distribution of the expected elements.

To further investigate the phase purity, powder X-ray diffraction (P-XRD) data were collected for all HE-DPs and the parent compound Cs_2_NaHoCl_6_ (Figure 1c, d). This comparison reveals a distinct shift of the diffraction peaks toward higher angles for HE-DPs compared to Cs_2_NaHoCl_6_. This observation, consistent with the single-crystal XRD results, indicates a shrinkage of the unit cell volume upon alloying. The lattice shrinkage is primarily attributed to the incorporation of substituent elements possessing smaller ionic radii (i.e., In^3^^+^ and Sb^3^^+^) into the perovskite framework. Importantly, the P-XRD patterns exhibit sharp diffraction peaks corresponding to the double perovskite phase with no discernible impurity phases, confirming the successful synthesis of phase-pure high-entropy crystals. Fine scans were also performed to identify the single-phase crystal system for HE-DPs, which displayed a good fit with a single Lorentzian function over the prominent (220) FCC reflection for all HE-DPs (Figure 1d). Furthermore, a notable reduction in the relative intensity of the (111) superstructure reflection is observed in **AgNaInSbHoErBi** compared to that of Cs_2_NaHoCl_6_. Since the intensity of this reflection is directly related to the degree of long-range ordering between the M^I^ and M^I^ ^I^ ^I^ cations, its attenuation suggests an increased B-site cation disorder in the high-entropy system.^43^ This likely arises from a higher concentration of antisite defects, where M^I^ (Na/Ag) and M^III^ (Ln/In/Sb/Bi) cations partially swap positions, thereby disrupting the ideal alternating M^I^-M^III^ sublattice arrangement.

### Entropy-Enabled Phase Stabilization

One major challenge for the lanthanide double perovskites is their phase instability, particularly upon exposure to humidity, light, and oxygen. Here, we propose that entropy engineering stabilizes the phase of lanthanide double perovskites by lowering the overall formation energy. To test this, we synthesized medium-entropy double perovskite, namely, Cs_2_Na­{InSbHo}_1_Cl_6_ (**InSbHo**). We then monitored the air stability of double perovskites with low- (Cs_2_NaHoCl_6_), medium- (**InSbHo**), and high-entropy (**AgNaInSbHoErBi**) compositions by P-XRD (Figure 2a). All the samples were ground into powder and stored under controlled conditions (20 °C, 70% relative humidity, and air atmosphere). Low-entropy lanthanide double perovskites, such as Cs_2_NaErCl_6_ or Cs_2_NaHoCl_6_, exhibit poor stability in air. Specifically, Cs_2_NaErCl_6_ loses its crystallinity within 2 days, while Cs_2_NaHoCl_6_ decomposes into HoCl_3_·6H_2_O within 5 days. In contrast, medium-entropy double perovskite (**InSbHo**) displays enhanced stability up to 9 days, while high-entropy double perovskite (**AgNaInSbHoErBi**) maintains its pristine crystal structure for about 90 days under otherwise identical conditions. The ambient stability of other medium- and high-entropy compositions was also tested, revealing a clear dependence on the specific combination of lanthanide ions (Figure S5). For clarity, we focus our discussion on representative systems containing Ho^3+^ and Er^3+^ ions to illustrate the correlation between the configurational entropy and stability. DPs with other Ln^3+^ cations (i.e., Yb^3+^, Tb^3+^, and Dy^3+^) display different stability trends owing to the distinct chemical stabilities associated with Ln^3+^. Our results clearly show a positive relationship between ambient stability and configurational entropy (Δ*S*_config_) (Figure 2b). To further exclude the formation of oxychlorides in these DPs, we performed room temperature Raman spectroscopic measurements (Figure S6), which show no evidence of O or OH.^44,45^ Therefore, our results highlight entropy engineering as a powerful strategy in designing unconventional and metastable solid-state materials.

**Figure 2:**
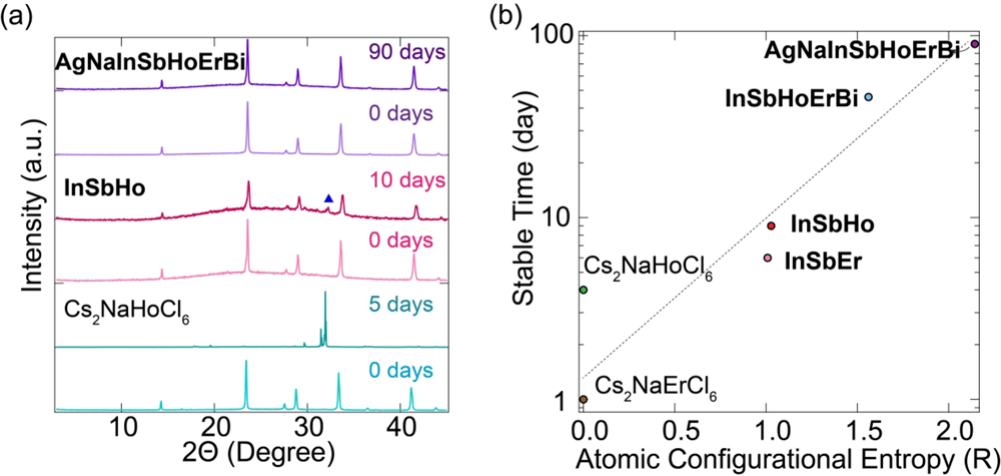
(a) P-XRD patterns for Cs_2_NaHoCl_6_, InSbHo, and AgNaInSbHoErBi. (b) Plot of stable time as a function of atomic configurational entropy.

### Downconverted Photoluminescence Modulated by Energy Flow Dynamics

To investigate the impact of compositional variances on the optoelectronic properties of double perovskites, we measured UV–vis absorption and photoluminescence (PL) spectra. The pristine Cs_2_NaInCl_6_ host exhibits a wide bandgap of approximately 4.4 eV. Upon incorporation of Sb^3+^ (10%), a distinct absorption band appears at ∼330 nm, which is attributed to the characteristic ^1^ *S*_0_ → ^3^ *P*_*n*=0,1,2_ electronic transition within the [SbCl_6_]^3–^ octahedra. Additionally, weaker absorption features are present near 400 nm, which can be assigned to defect states that facilitate the formation of self-trapped excitons (STEs).^46^ Further alloying with lanthanide (Ln^3+^) ions on the M^3+^ site introduces new absorption peaks at longer wavelengths, such as 450 and 520 nm (Figure S7), corresponding to the characteristic *f*–*f* transitions of the incorporated Ln^3+^ ions. The optical bandgap extracted from the Tauc plot of HE-DP **AgNaInSbHoErBi** is 2.82 eV, which is significantly narrowed compared to Cs_2_NaInCl_6_.

The PL spectra of the HE-DPs were first measured by excitation at 330 nm (Figure 3 and Figure S8). Consistent with previous reports, the Sb^3+^-doped Cs_2_NaInCl_6_:Sb exhibits a broad blue emission centered at 440 nm, attributed to the triplet-to-singlet (^3^ *P*_*n*=0,1,2_ → ^1^ *S*_0_) radiative recombination of STE from Sb^3+^ (5*s* ^2^) centers (Figure 3a). This suggests an efficient energy transfer pathway from the host to the Sb^3+^ dopants (Figure 3b). Remarkably, the PL profile is dramatically altered within the HE-DPs. Two distinct behaviors emerge in HE-DPs, namely, dominant *f*–*f* emissions and modulated broadband STE emission, both arising from the incorporation of Ln^3+^ ions. Taking **AgNaInSbHoErBi** as the representative example (Figure 3c), characteristic, sharp *f*–*f* emissions from Ho^3+^ (indicated by blue text) (^5^F_3_ → ^5^I_8_ (488 nm), ^5^F_4_ → ^5^I_8_ (550 nm), ^5^F_5_ → ^5^I_8_ (655 nm), ^5^I_5_ → ^5^I_8_ (905 nm), and ^5^F_5_ → ^5^I_7_ (1000 nm)) and Er^3+^ (indicated by green text) (^4^S_3/2_ → ^4^I_15/2_ (550 nm), ^4^I_9/2_ → ^4^I_15/2_ (800 nm)) are clearly observed and dominate the spectrum. Interestingly, all Ln^3+^ emissions, including those in NIR regimes (e.g., 1000 nm (NIR-I), arising from the ^2^F_5/2_ → ^2^F_7/2_ transition of Yb^3+^, and 1540 nm (NIR-II) from ^4^I_13/2_ → ^4^I_15/2_ of Er^3+^), can be clearly observed in the corresponding HE-DP (Figure 3, Figure S8). The relative photoluminescence quantum yield (PLQY) of the NIR-II emission in **AgNaInSbHoErBi** was measured as 8.7% (Table S5), underscoring the potential of HE-DPs for efficient NIR-II emitters. Additionally, the broad STE emission is found to be substantially quenched in **AgNaInSbHoErBi**. A similar dominance of *f*–*f* emission is also observed in other compositions such as **InSbHoErYb** and **InSbHoErBi**. The quenched STE emission from the Sb^3+^ centers suggests an energy transfer from STE states to Ln^3+^ ions (Figure 3d).

**Figure 3:**
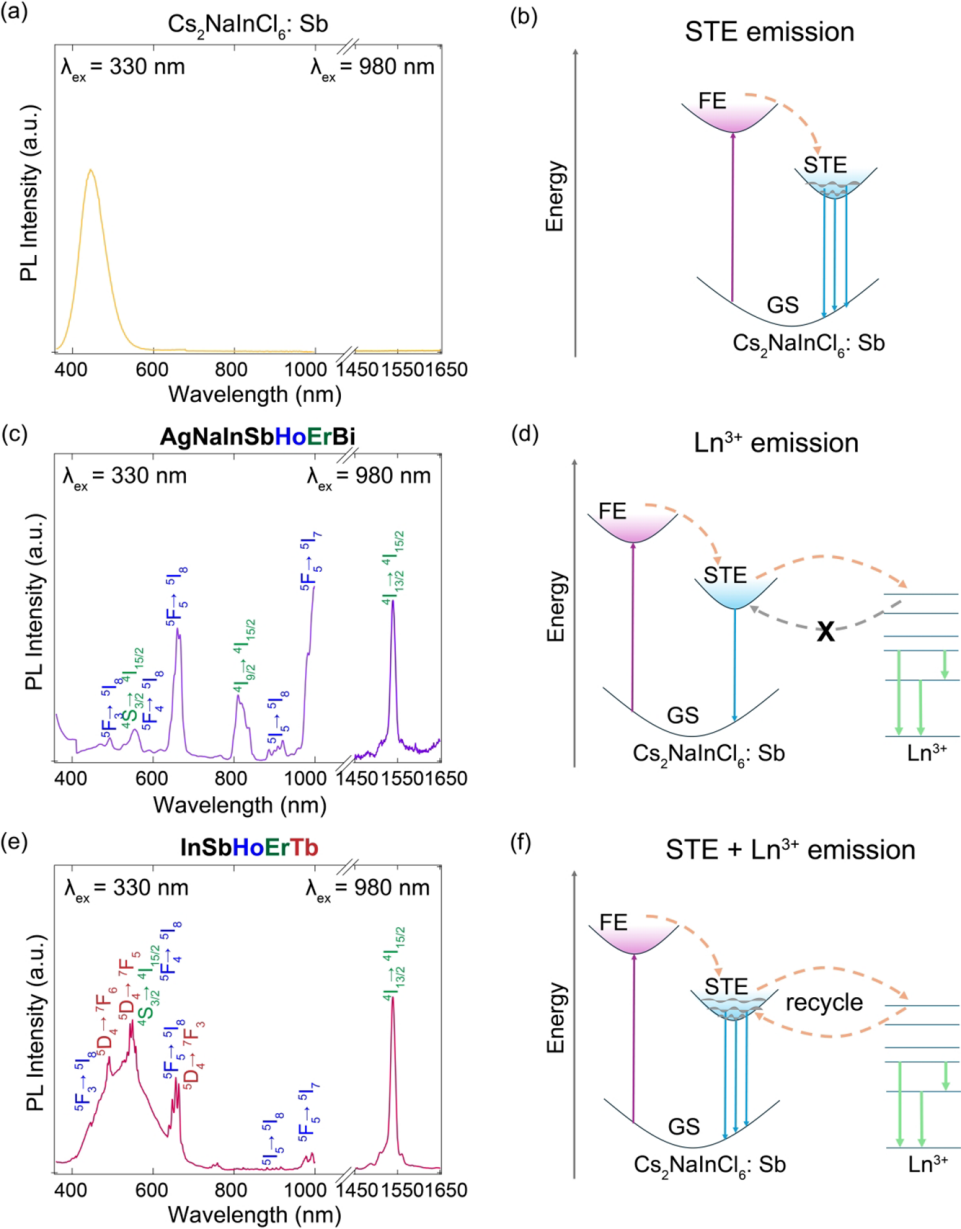
PL spectra of (a) Cs_2_NaInCl_6_:Sb, (b) AgNaInSbHoErBi, and (c) InSbHoErTb. The proposed luminescence mechanism for (d) Cs_2_NaInCl_6_:Sb, (e) AgNaInSbHoErBi, and (f) InSbHoErTb.

To verify the proposed mechanism, we measured the photoluminescence excitation (PLE) spectra by monitoring both the STE emission (440 nm) and the characteristic Ho^3+^ and Er^3+^ emission (550 nm), as shown in Figure S9. The PLE spectrum of the STE emission features a broad excitation band around 318 and 335 nm, originating from the ^1^ *S*_0_ → ^3^ *P*_*n*=0,1,2_ transition of Sb^3+^. Crucially, the PLE spectra for the Ln^3+^ emissions mirror this profile, showing dominant excitation via the same Sb^3+^-related band, while direct *f*–*f* excitation contributions are minimal. This provides direct evidence that Ln^3+^ luminescence is primarily sensitized through efficient energy transfer from the host-generated STEs rather than direct absorption.

Interestingly, compositions such as **InSbHoErTb** showcase a different phenomenon (Figure 3e). In addition to the *f*–*f* transitions from Ho^3+^, Er^3+^, and Tb^3+^ (indicated by red text) (^5^D_4_ → ^7^F_6_ (490 nm), ^5^D_4_ → ^7^F_5_ (544 nm), and ^5^D_4_ → ^7^F_3_ (622 nm)), this composition exhibits a significantly broadened and red-shifted STE emission band that covers nearly the entire visible spectrum from 400 to 760 nm. A similar trend is observed upon replacing Tb^3+^ with Dy^3+^, namely, in **InSbHoErDy** (Figure S8). The intriguing broadening and enhancement of the STE emission, which is not observed in simpler doped systems,^16,17,47^ point toward a unique mechanism unlocked by the high-entropy design. The observed STE emission suggests that the outflow energy can be recycled back to the STE states (Figure 3f), while the spectral broadening can be ascribed to an enhanced electron–phonon coupling owing to the increased lattice distortion from the high-entropy nature.^48,49^ Specifically, the incorporation of multiple cations at high concentrations, including main group elements (In^3+^, Bi^3+^, Sb^3+^) and lanthanides (Ho^3+^, Er^3+^, Yb^3+^, Dy^3+^, Tb^3+^) with large disparities in the ionic radii, creates a highly disordered local environment.

To extract the electron–phonon coupling strength, we conducted a temperature-dependent PL study on Cs_2_NaInCl_6_:Sb and **InSbHoErTb** over the temperature range from 100 to 340 K. The Huang–Rhys factors, obtained by fitting the temperature-dependent PL full width at half-maximum (fwhm), were calculated as 6.56 and 26.5 for Cs_2_NaInCl_6_: Sb and **InSbHoErTb**, respectively (Figure S10). A larger Huang–Rhys factor indicates stronger electron–phonon coupling in the HE-DPs, which is consistent with their broadened STE emission in **InSbHoErTb**.

To further understand the PL mechanism and energy transfer dynamics in these HE-DP, time-resolved PL (TRPL) measurements were performed (Figure S11). The dynamics of the STE emission (monitored at 440 nm) are characterized by decay lifetimes in the microsecond (μs) range (Figure S11a and Table S6), consistent with triplet state recombination. In contrast, the sharp *f*–*f* emissions (e.g., monitored at 550 nm for Er^3+^) exhibit substantially longer decay lifetimes, reaching the millisecond (ms) scale. This order-of-magnitude difference is a signature of the Laporte-forbidden *f*–*f* transitions within the Ln^3+^ ions. However, it is noteworthy that both the lifetimes and intensities of these *f*–*f* emissions in our HE-DPs are generally lower than those in conventional singly doped systems. This suggests a dynamic energy flow between Ln^3+^ excited states and STE states, as well as other nonradiative decay channels, such as energy migration among Ln^3+^ ions followed by cross-relaxation.^50,51^

To rationalize the pathways between Figure 3d and f, we examine the compositional difference in HE-DPs. Notably, the HE-DPs that display both STE and *f*–*f* transitions appear to possess a higher content of Sb^3+^ compared to those that only show *f*–*f* transitions. On the other hand, for systems with a high total lanthanide content, such as **InSbHoErYb** where the total Ln^3+^ ratio is approximately 70%, only *f*–*f* transitions were observed in the PL spectra. Therefore, we propose that there is a dynamic competition for energy flow between Ln^3+^ and STE states (based on Sb^3+^ ions). According to Fermi’s golden rule, the transition rate between two states is proportional to the density of acceptor states. In the case of HE-DPs with a high total lanthanide content, the energy flow from STE states to Ln^3+^ states is expected to be much faster than that with a lower total lanthanide content, resulting in a quenched STE emission. On the other hand, when the Sb^3+^ content is higher, the STE state can simultaneously undergo radiative recombination and energy migration, giving rise to both STE and *f*–*f* transitions. We envision that there could also be energy recycling between Ln^3+^ and STE states (*vide infra*) owing to the ion proximity in the HE-DP systems. Therefore, HE alloying clearly presents a viable and elegant approach to tune the PL properties of halide perovskites by modulating the multichannel energy flow dynamics, and the precise elemental composition of HE-DP is the key to the different radiative pathways.

### Upconverted Photoluminescence Modulated by the Proximity Effect

Inspired by the dynamic energy flow observed between Ln^3+^ and STE states, we hypothesize that the multicomponent, proximity Ln^3+^ and Sb^3+^/Bi^3+^ ions can also interact efficiently to trigger upconverted PL.^15,52,53^ For instance, the Yb^3+^ ion is wildly used as a sensitizer with a sufficient absorption cross-section in the NIR region, while at the same time, its ^2^F_7/2_ → ^2^F_5/2_ transition is well resonant with many *f*–*f* transitions of typical upconverting lanthanide ions (e.g., Er^3+^, Tm^3+^, and Ho^3+^). Tremendous progress has been made in colloidal nanocrystals codoped with Yb^3+^/Er^3+^ ions exhibiting NIR-to-visible photon upconversion;^54,55^ however, it remains nontrivial to design host materials that can promote the energy transfer between dopants without severe cross-relaxation loss. Here, we hypothesize that the HE-DP offers an ideal platform to codope various Ln^3+^ ions for harvesting upconversion luminescence. To test this, we perform PL measurements of HE-DP under 980 nm laser excitation. Interestingly, one HE-DP, namely, **InSbHoErYb**, which contains Yb^3+^ (sensitizer) and Er^3+^/Ho^3+^ (activator), displays intense green upconverted emission (Figure 4a). To further validate the proposed parallel upconversion mechanisms, power-dependent PL was measured by monitoring the intense emission peak at 550 nm (Figure 4b), which comprises overlapping contributions from both Er^3+^ (^4^S_3/2_ → ^4^I_15/2_) and Ho^3+^ (^5^S_2_ → ^5^I_8_) transitions. At low power densities, the integrated emission intensity shows a quadratic dependence on the excitation power, confirming a two-photon process. As the power increases, this dependence shifts to a linear trend, indicating the saturation of intermediate excited states, a classic signature of energy transfer upconversion (ETU).^56^

**Figure 4:**
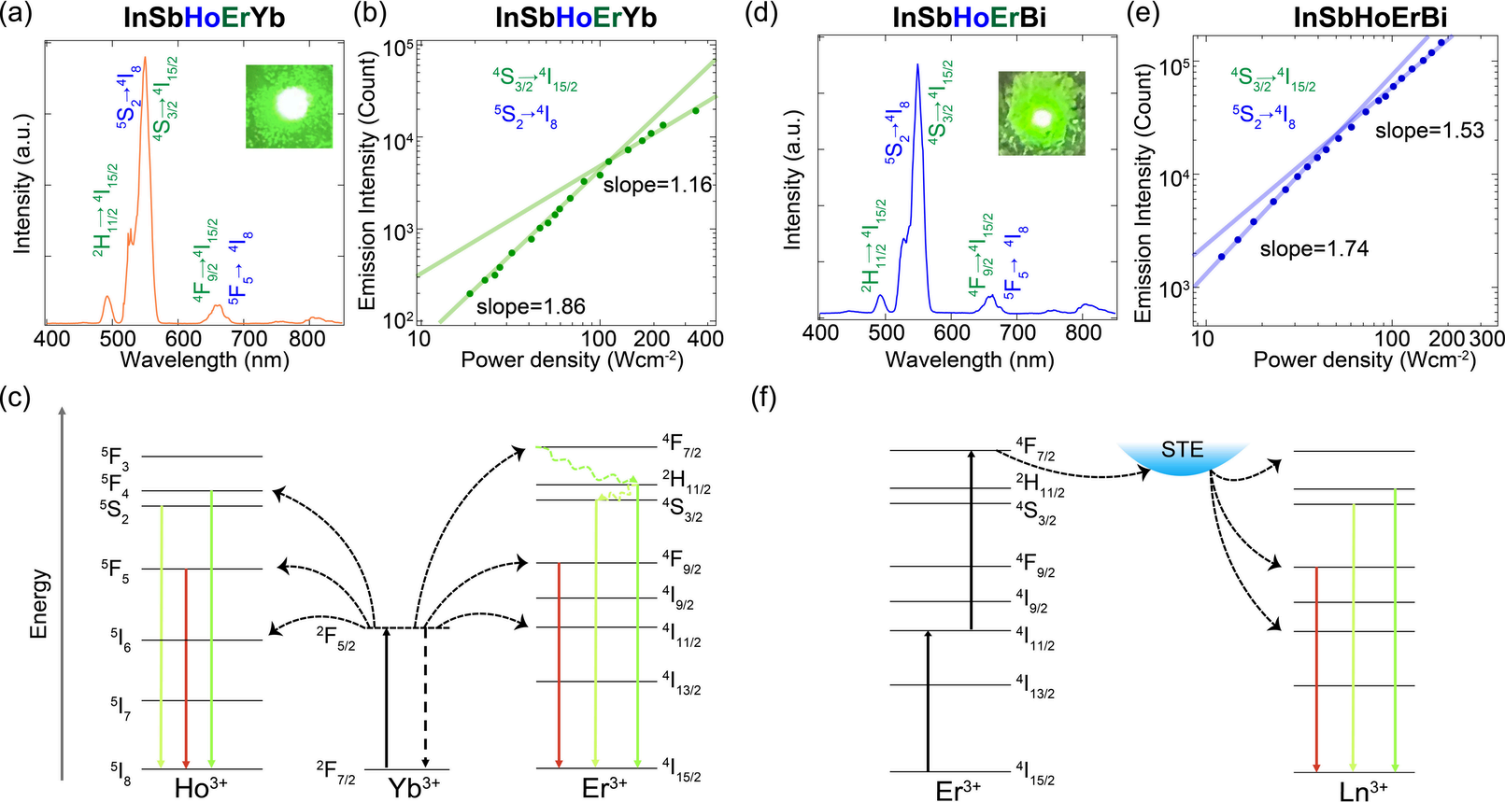
(a) Upconversion emission for the InSbHoErYb. The inset is a photograph of NIR-to-visible upconversion. (b) Power dependent UC emission and (c) proposed upconversion luminescence mechanism for InSbHoErYb. (d) Upconversion emission for the InSbHoErBi. The inset is a photograph of NIR-to-visible upconversion. (e) Power dependent UC emission and (f) proposed upconversion luminescence mechanism for InSbHoErBi.

The underlying mechanism for the observed upconversion luminescence involves parallel ETU pathways, with Yb^3+^ acting as the sensitizer and both Er^3+^ and Ho^3+^ serving as activators (Figure 4c). Upon excitation at 980 nm, a Yb^3+^ ion absorbs a photon and is promoted from the ^2^F_7/2_ ground state to the ^2^F_5/2_ excited state. This energy is then transferred sequentially to the activators. For Er^3+^, two consecutive energy transfers promote it from the ^4^I_15/2_ ground state to the ^4^I_11/2_ state and subsequently to the ^4^F_7/2_ level. Nonradiative relaxations then populate the ^2^H_11/2_, ^4^S_3/2_, and ^4^F_9/2_ states, leading to the characteristic green and red emissions. Concurrently, a similar two-step ETU process excites the Ho^3+^ ion from its ^5^I_8_ ground state, through the ^5^I_6_ intermediate state, to the ^5^F_4_ and ^5^S_2_ emitting levels. Radiative decay from these states (and the ^5^F_5_ state populated via relaxation) also contributes significantly to the green and red emission bands. Therefore, the overall intense visible luminescence is a superposition of radiative transitions from both Er^3+^ and Ho^3+^ ions, enabled by the efficient ETU from Yb^3+^.

A more striking and unconventional mechanism emerged in compositions where Yb^3^^+^ was substituted with Bi^3+^, such as that in **InSbHoErBi** (Figure 4d). Despite lacking a conventional sensitizer, this material displayed bright green UC emission initiated by Er^3+^ ions acting as the primary absorbers of the 980 nm excitation. Notably, **AgNaInSbHoErBi**, also containing Bi^3+^ but not Yb^3+^, similarly exhibits upconversion emission (Figure S12), suggesting the crucial role of Bi^3+^ in facilitating this process. Inspired by recent reports,^13,57^ we postulate a dual role for Bi^3+^ (Figure 4f): it not only facilitates the formation of host STE states that serve as energy-transfer mediators but also plays a crucial role in modulating the electronic structure of the host lattice. This lattice modulation is proposed to establish an efficient energy transfer channel, possibly via sub-bandgap states, that becomes energetically resonant with the sequential electronic transitions within the Ln^3+^ activators. This Bi^3+^-mediate pathway substantially enhances the otherwise inefficient ETU process. Additionally, Bi^3+^ functions as a diluent ion to increase the Ln^3+^-Ln^3+^ distance, which suppresses concentration quenching and undesirable cross-relaxation that would otherwise depopulate the emissive states. The power-dependent measurement for **InSbHoErBi** (Figure 4e) confirms a two-photon UC process responsible for the emission. The observation of upconversion is not a general feature of all of the HE-DPs. For instance, compositions like **InSbHoErDy** and **InSbHoErTb** were found to display zero upconversion PL. This suggests that the presence of specific ions, namely, Yb^3+^ or Bi^3+^, is essential to enable the UC process. This discovery of two distinct UC pathways within a single class of materials underscores the versatility of the high-entropy strategy. Our HE-DP systems present a unique and stable halide perovskite system that displays multichannel and highly tunable PL properties.

## Conclusions

In summary, we successfully synthesized and characterized a new series of high-entropy halide double perovskites via a mild hydrothermal method. By simultaneously introducing multiple elements (Ln^3+^, In^3+^, Sb^3+^, Bi^3+^, and Ag^+^) onto the parent Cs_2_NaHoCl_6_ structure, we validated the feasibility of the high-entropy strategy for stabilizing complex compositions within the double perovskite framework. These high-entropy materials demonstrated markedly improved ambient stability compared with their parent structure. More importantly, HE alloying gives rise to emerging optoelectronic properties that are otherwise unattainable in simple perovskites. In particular, they exhibit narrowed bandgap, tunable and multichannel emission characteristics, including broadband STE and sharp *f*–*f* emission under UV excitation, and upconversion luminescence under NIR excitation. This work underscores the potential of the high-entropy concept for designing robust halide double perovskites with tailored, multifunctional optical properties, opening new avenues for their application in advanced lighting, displays, and anticounterfeiting technologies.

## Experimental Section

### Methods

#### Materials

Unless otherwise noted, all chemicals were used as received. Cesium chloride (CsCl, 99.9%), bismuth oxide (Bi_2_O_3_, 99.9%), and antimony trioxide (Sb_2_O_3_, 99.9%) were purchased from Aladdin. Sodium chloride (NaCl, 99.99%) was purchased from Alfa Aesar. Silver oxide (Ag_2_O, 99%) was purchased from Dieckmann. Holmium chloride hexahydrate (HoCl_3_·6H_2_O, 99.9%), erbium chloride (ErCl_3_, 99.9%), dysprosium chloride (DyCl_3_, 99.99%), terbium chloride (TbCl_3_, 99.99%), ytterbium chloride (YbCl_3_, 99.9%), and indium­(III) oxide (In_2_O_3_, 99.99%) were purchased from Macklin. Hydrochloric acid (HCl, 37%) was purchased from Sigma-Aldrich.

#### Synthesis of Sb^3+^ Alloyed Cs_2_NaInCl_6_ Single Crystals

A hydrothermal method was used to synthesize Sb^3+^-alloyed Cs_2_NaInCl_6_ single crystals. First, 1 mmol of CsCl, 0.5 mmol of NaCl, 0.25 mmol of In_2_O_3_, 0.05 mmol of Sb_2_O_3_, and 10 mL of 12 M hydrochloric acid were loaded in a 25 mL Teflon liner. The solution was heated at 180 °C for 12 h in a stainless-steel Parr autoclave and then cooled to room temperature. Single crystals were isolated by vacuum filtration.

#### Synthesis of (Sb^3+^, Ln^3+^) Alloyed Cs_2_NaInCl_6_ Single Crystals

The synthesis process was similar to that of Sb^3+^-alloyed Cs_2_NaInCl_6_ single crystals. The only difference was that the Sb_2_O_3_, In_2_O_3_, and LnCl_3_ contents were fixed at 0.02, 0.05, and 0.1 mmol, respectively.

#### Synthesis of (Sb^3+^, Bi^3+^, Ln^3+^) Alloyed Cs_2_NaInCl_6_ Single Crystals

The synthesis process was similar to that of Sb^3+^, Ln^3+^-alloyed Cs_2_NaInCl_6_ single crystals. The only difference was that the Sb_2_O_3_, Bi_2_O_3_, In_2_O_3_, and LnCl_3_ contents were fixed at 0.02, 0.03, 0.05, and 0.1 mmol, respectively.

#### Synthesis of (Ag^+^, Sb^3+^, Bi^3+^, Ln^3+^) Alloyed Cs_2_NaInCl_6_ Single Crystals

The synthesis process was similar to that of Sb^3+^, Bi^3+^, and Ln^3+^-alloyed Cs_2_NaInCl_6_ single crystals. The only difference was that the NaCl and Ag_2_O contents were fixed at 0.25 and 0.125 mmol, respectively.

#### Single Crystal X-ray Diffraction and Powder X-ray Diffraction

Single crystal X-ray diffraction was conducted on a Bruker Incoatec ImuS Ag Diamond II with a PHOTO III diffractometer. The crystal was kept at 173.15 K during the data collection. Using Olex2, the structure was solved with the SHELXT structure solution program using Intrinsic Phasing and refined with the SHELXL refinement package using Least Squares minimization.

Powder X-ray diffraction data were collected on a Rigaku MiniFlex powder-X-ray diffractometer with a Cu source.

#### UV–Visible Spectroscopy

Diffuse reflectance spectra were obtained by using a UH5700 spectrophotometer equipped with a 60 mm diameter integrating sphere. The crystal samples were ground and diluted with barium sulfate. The diffuse reflectance spectra were measured from 200 to 1000 nm. The Kubelka–Munk equation was then used to convert the reflectance to the absorption data: α/*S* = (1 – *R*)^2^/(2*R*), where *R* is the reflectance, α is the absorption coefficient, and *S* is the scattering coefficient. Bandgaps of the double perovskite were calculated by the Tauc plot.

#### Energy-Dispersive X-ray Spectroscopy

The distribution of elemental composition of the materials was determined by energy-dispersive X-ray spectroscopy analysis, which was incorporated with scanning electron microscopy analysis. The test was performed with powder samples using a JSM-6390 (JEOL).

#### Inductively Coupled Plasma–Optical Emission Spectrometer (ICP–OES)

Elemental composition was determined using a PerkinElmer AVIO-200 inductively coupled plasma-optical emission spectrometer. Prior to testing, the crystal samples were ground and then dissolved in a dilute acid solution.

#### Photoluminescence Spectroscopy

The PL spectra of visible and NIR were collected by a NOVA spectrometer through a confocal microscope and were excited by a 330 nm laser. The PL spectra of upconversion and near-infrared spectra were obtained with a Horiba Fluorolog-3 spectrofluorometer by a 980 nm laser.

Temperature-dependent PL emission spectra were measured from 100 to 340 K in a cryostat cooled by liquid nitrogen. The sample was excited by a 330 nm cw-laser. The full width at half-maximum (fwhm) was extracted from the measured spectra at each temperature. The Huang–Rhys factor and phonon frequency of the sample were derived from the following equation:

$$\mathrm{FWHM}=2.36\sqrt{\left(S\right)}\hbar \omega_{\mathrm{Phonon}}\sqrt{\left(\coth\;\left[\frac{\left(\hbar \omega_{\mathrm{Phonon}}\right)}{\left(2k_{B}T\right)}\right]\right)}$$

where *S* is the Huang–Rhys factor, ω_Phonon_ is the phonon frequency, *T* is the temperature (K), and *k*_B_ is the Boltzmann constant.

#### Photoluminescence Quantum Yield (PLQY)

PLQY measurements were recorded by using a HAMAMATSU UV-NIR absolute PL quantum yield spectrometer. The excitation source was provided by 808 or 365 nm laser setups.

#### Time-Resolved Photoluminescence (TRPL)

Time-resolved PL (TRPL) measurements were recorded using an Edinburgh Instruments FLS 980.

#### Raman Spectroscopy

Raman spectra were collected by using a Renishaw inVia micro-Raman system with a 785 nm diode laser as the excitation source. The spectral range was measured from 200 to1000 cm^–1^.

## Supplementary Material



## Notes

Structural characterization, SEM data, and additional spectroscopic data (PDF)
