# Supplementary material for: High-Entropy Double Perovskites with Tailored Multichannel Luminescence
Source: J Am Chem Soc. 2025 Dec 11;147(51):47764–72. doi: 10.1021/jacs.5c18097 (PMC12752456; doi:10.1021/jacs.5c18097)
Supplement: Supplementary file 1 [file ja5c18097_si_001.pdf]

Supporting Information

High-Entropy Double Perovskites with Tailored Multichannel Luminescence

Jie Xue,[1] Jun Luo,[2] Kin Ting Chang,[1] Zihang Sun,[1] Zonglong Zhu,[3] Lingling Mao*,[2] Haipeng Lu*[1]

[1] Department of Chemistry, The Hong Kong University of Science and Technology, Clear Water Bay, Kowloon, Hong Kong, 999077 China (SAR)

[2] Department of Chemistry, Southern University of Science and Technology, Shenzhen, Guangdong, 518055 China

[3] Department of Chemistry, City University of Hong Kong, Kowloon, Hong Kong, 999077 China (SAR)

Corresponding Author

Lingling Mao*: maoll@sustech.edu.cn; Haipeng Lu*: haipenglu@ust.hk

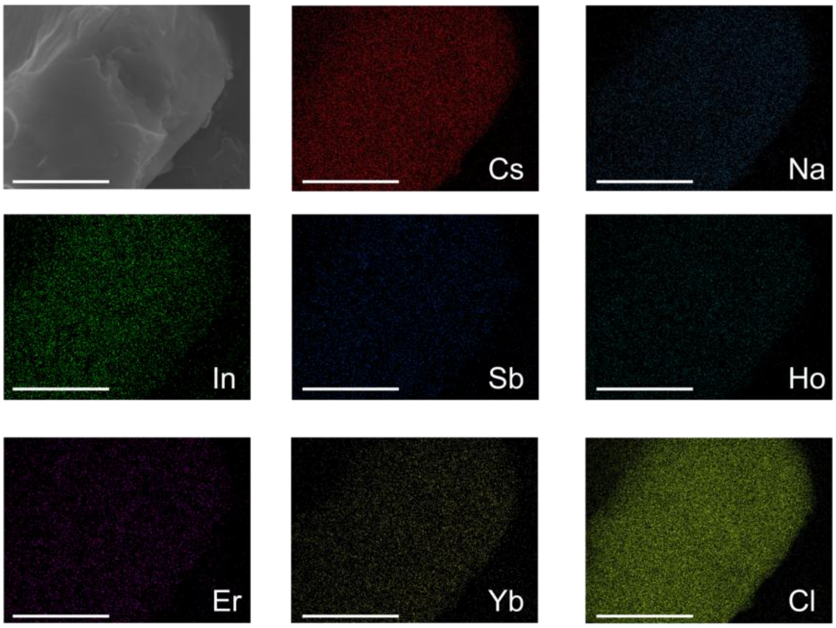

Figure S1. SEM-EDX results for the ground **InSbHoErYb** sample. The scale bar is 10 μm in the SEM images.

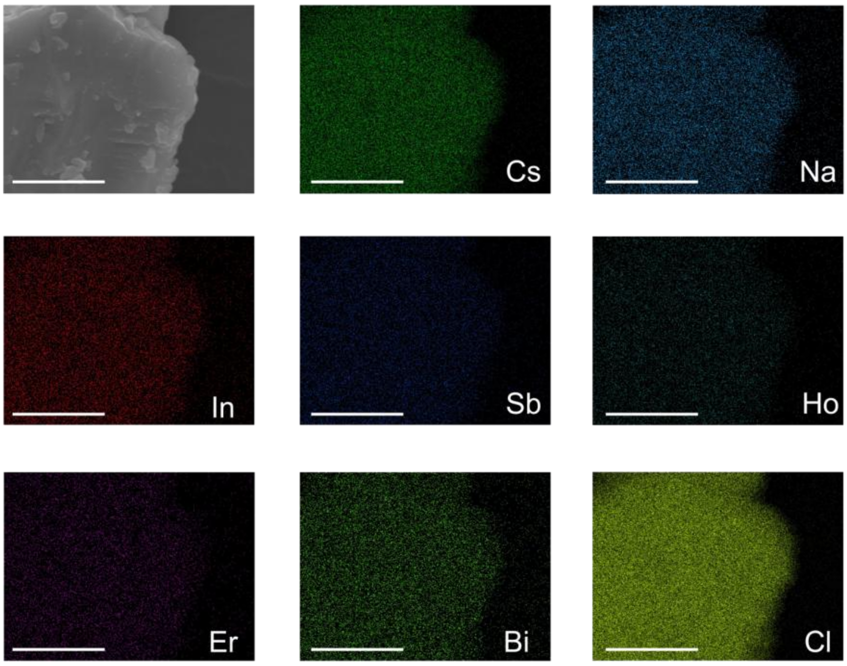

Figure S2. SEM-EDX results for the ground **InSbHoErBi** sample. The scale bar is 10 μm in the SEM images.

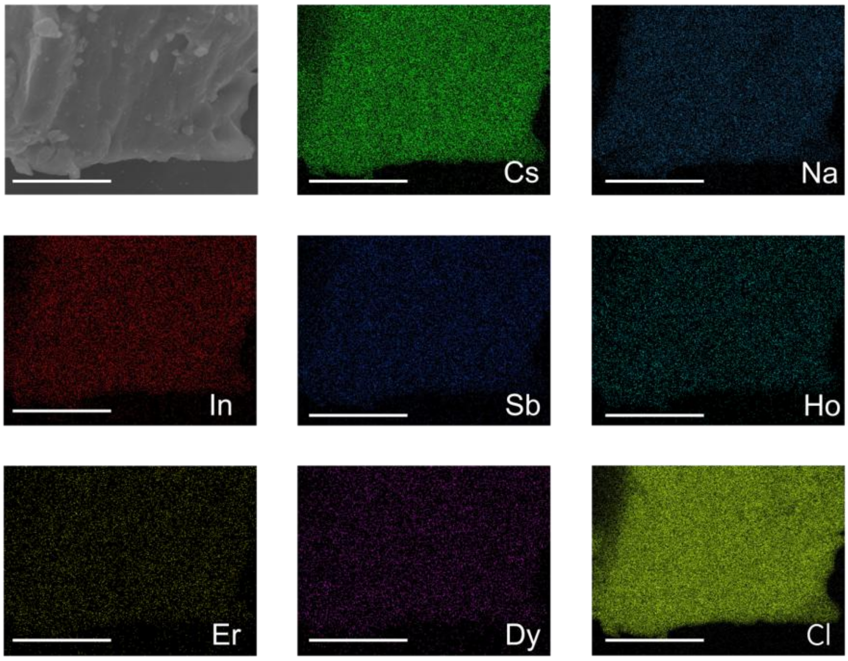

Figure S3. SEM-EDX results for the ground **InSbHoErDy** sample. The scale bar is 10 μm in the SEM images.

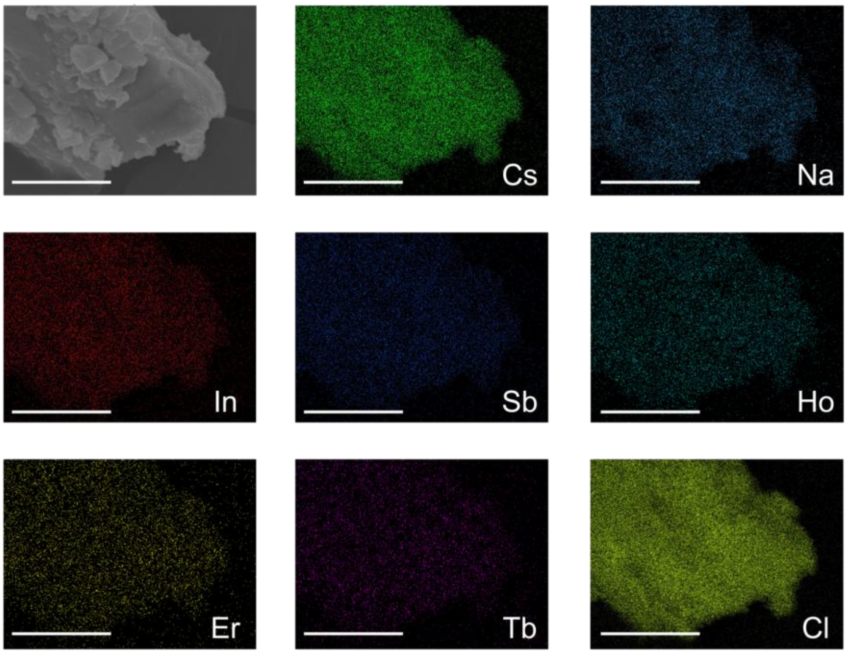

Figure S4. SEM-EDX results for the ground **InSbHoErTb** sample. The scale bar is 10 μm in the SEM images.

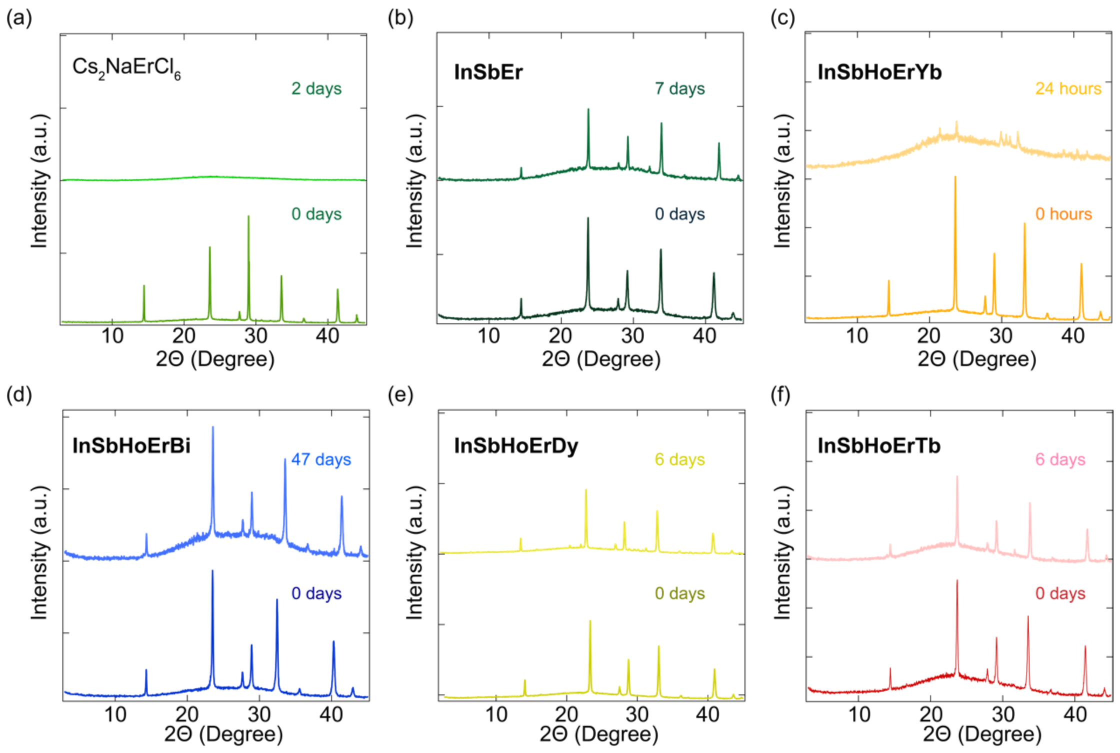

Figure S5. P-XRD patterns for (a) $Cs_2NaErCl_6$, (b)**InSbEr**, (c) **InSbHoErYb**, (d) **InSbHoErBi**, (e) **InSbHoErDy** and (f) **InSbHoErTb**. Note that different HE-DP crystals exhibit varying levels of air stability due to their differing hygroscopic properties.

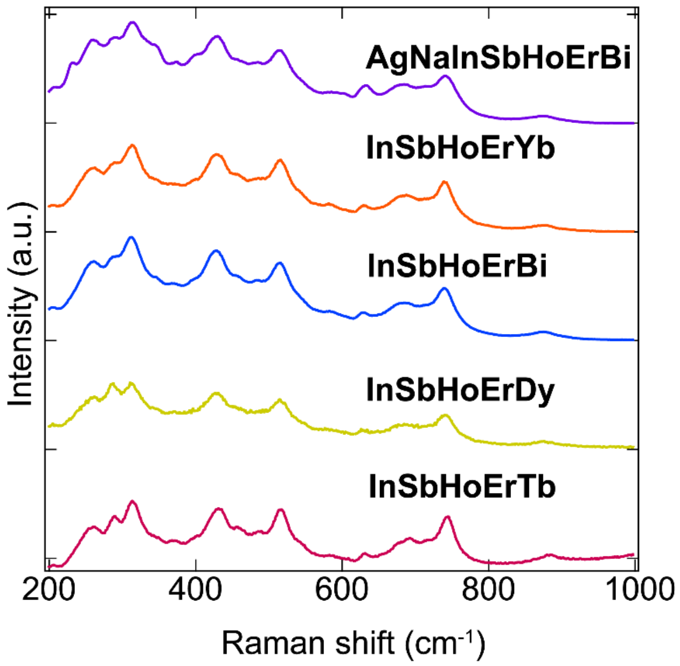

Figure S6. Room-temperature Raman spectra of high-entropy double perovskite crystals.

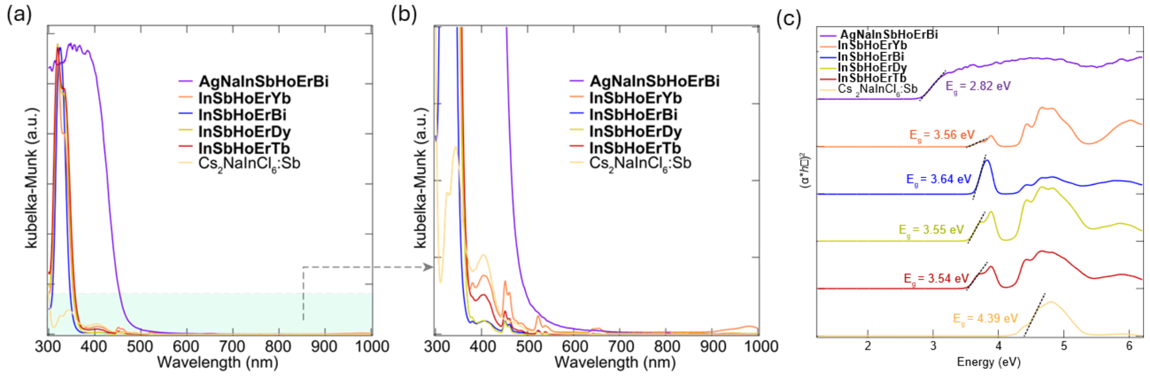

Figure S7. (a) The optical absorption spectrum, (b) magnified spectrum, and (c) Tauc plot of normal double perovskite and high-entropy double perovskites.

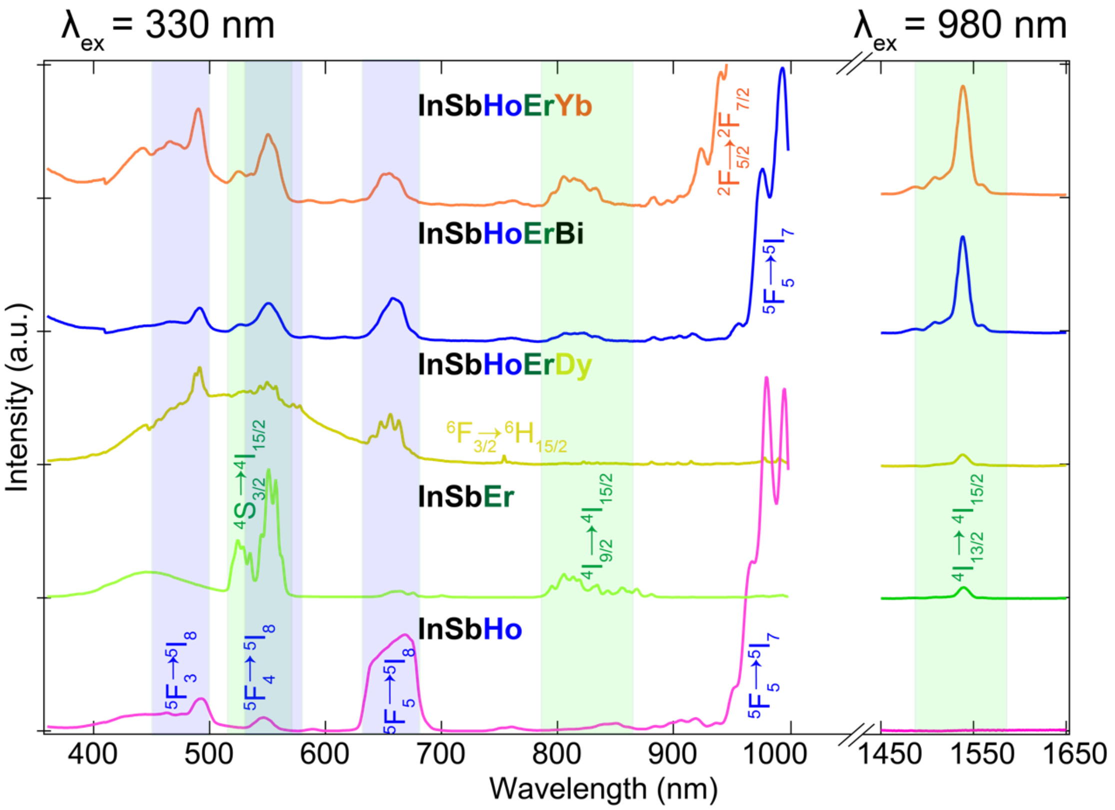

Figure S8. The PL spectra of **InSbHoErYb**, **InSbHoErBi**, **InSbHoErDy**, **InSbEr** and **InSbHo**.

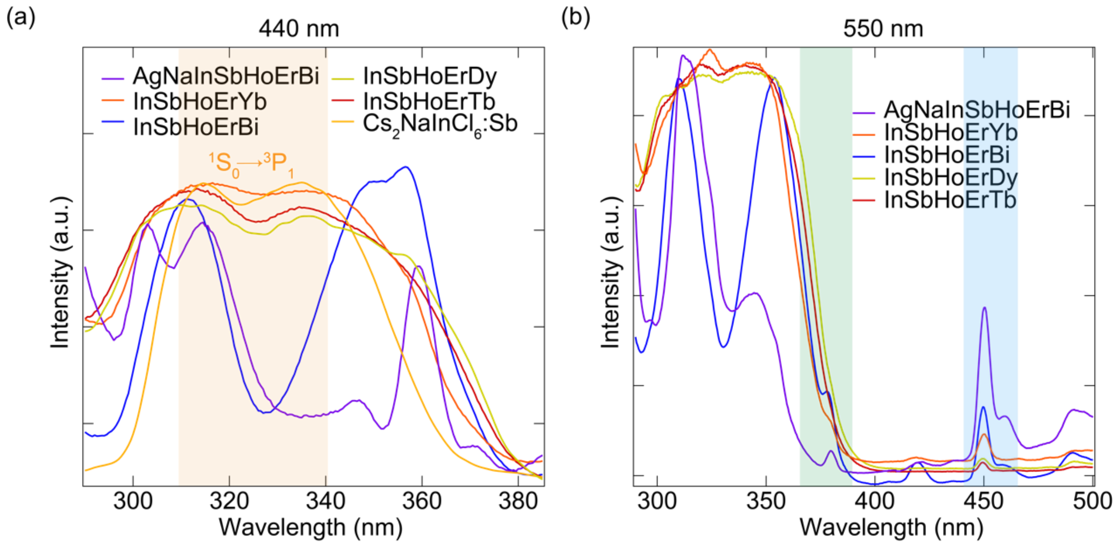

Figure S9. (a) PLE spectra of $Cs_2NaInCl_6$: Sb and HE-DPs at emission wavelength of 440 nm. (b) PLE spectra of HE-DPs at emission wavelength of 550 nm.

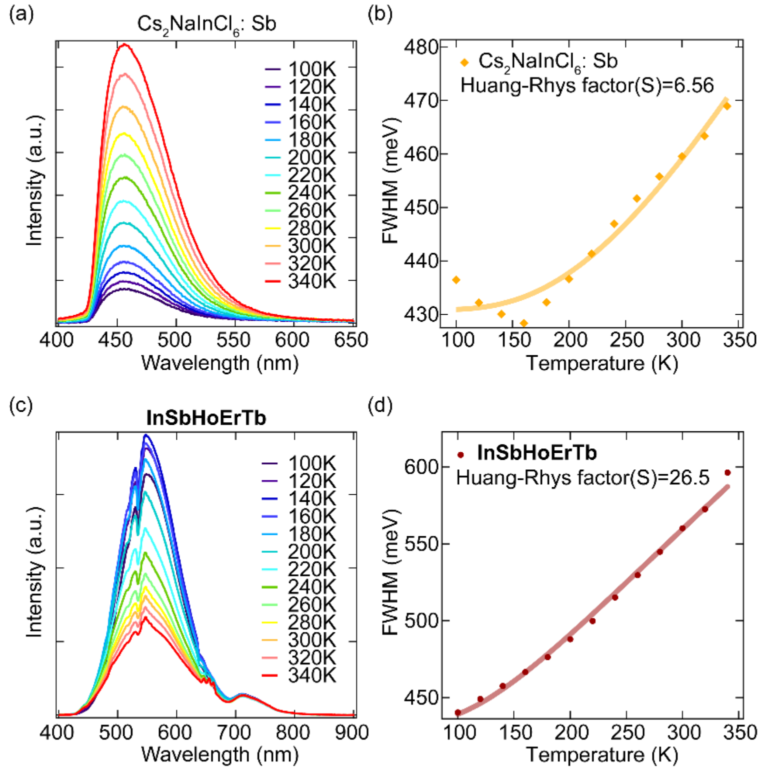

Figure S10. Temperature-dependent PL spectra and fitting results of the FWHM as a function of the temperature for $Cs_2NaInCl_6$: Sb (a-b) and **InSbHoErTb** (c-d) crystals.

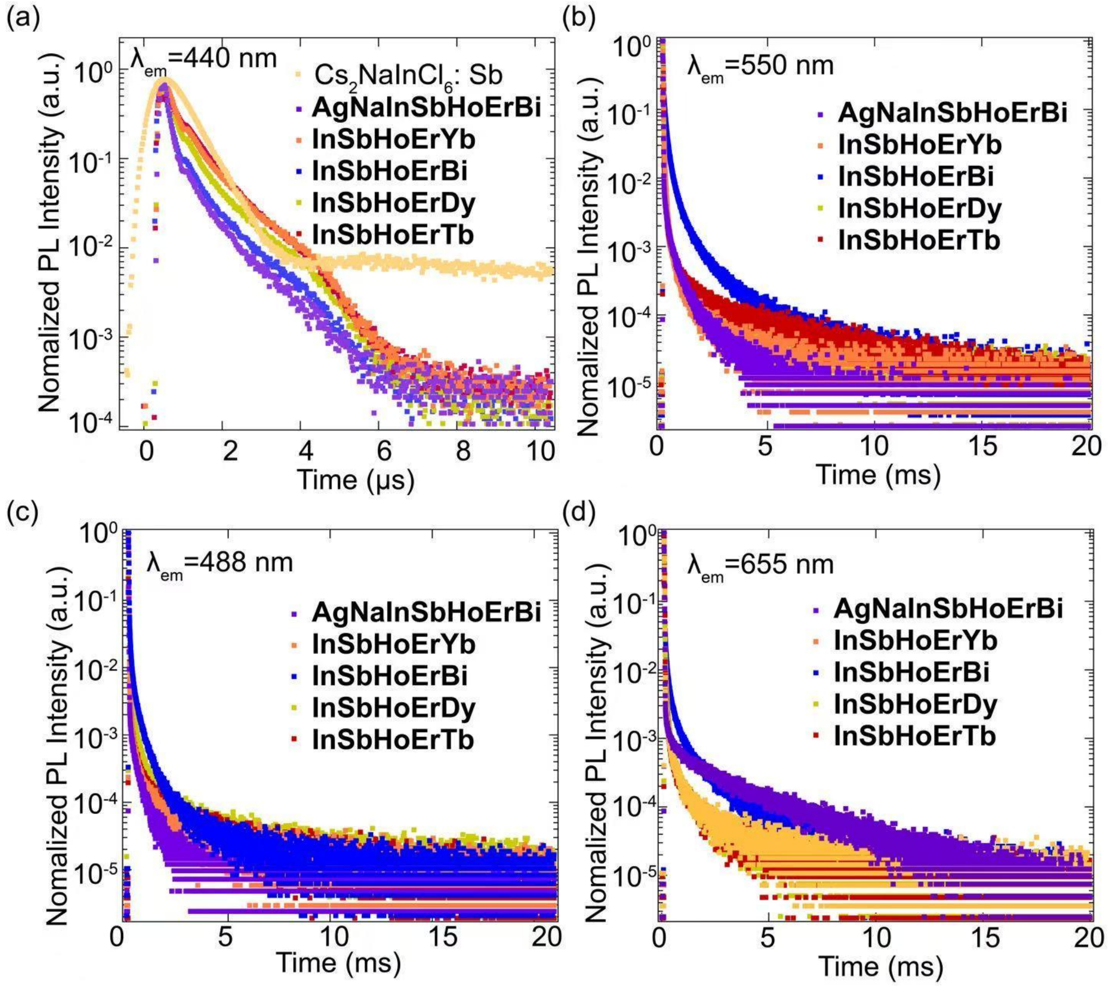

Figure S11. (a) Emission decay curves of $Cs_2NaInCl_6$: Sb and HE-DPs probed at 440 nm. (b) Emission decay curves of HE-DPs probed at 550 nm. (c) Emission decay curves of HE-DPs probed at 488 nm. (d) Emission decay curves of HE-DPs probed at 655 nm.

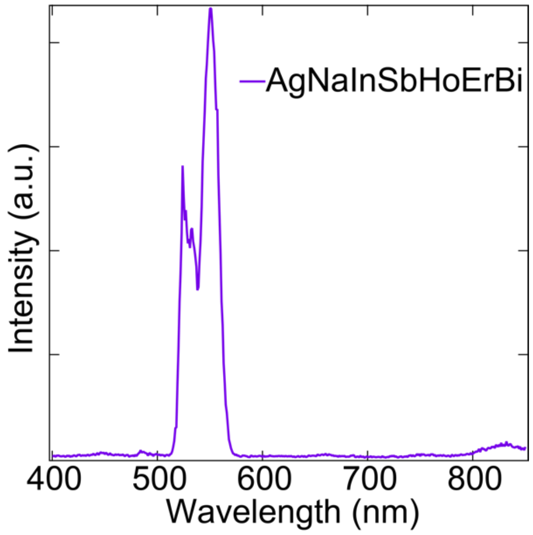

Figure S12. Up-conversion emission spectrum of **AgNaInSbHoErBi** crystal excited at 980 nm.

Table S1. ΔS_{config} and Inductively Coupled Plasma Optical Emission Spectrometry (ICP-OES) results for **AgNaInSbHoErBi**, **InSbHoErYb**, **InSbHoErBi**, **InSbHoErDy**, **InSbHoErTb, InSbHo and InSbEr**.

	Na	Ag	In	Sb	Ho	Er	Bi	Yb	Dy	Tb	ΔS/R
**AgNaInSbHoErBi**	0.67	0.33	0.33	0.22	0.13	0.08	0.24	/	/	/	2.12
**InSbHoErYb**	1	/	0.25	0.07	0.28	0.14	/	0.26	/	/	1.51
**InSbHoErBi**	1	/	0.30	0.15	0.25	0.14	0.16	/	/	/	1.56
**InSbHoErDy**	1	/	0.33	0.11	0.28	0.15	/	/	0.13	/	1.51
**InSbHoErTb**	1	/	0.26	0.15	0.28	0.17	/	/	/	0.14	1.57
**InSbHo (M. E.)**	1	/	0.48	0.18	0.34	/	/	/	/	/	1.03
**InSbEr (M. E.)**	1	/	0.53	0.19	/	0.28	/	/	/	/	1.01

Table S2. Crystal data and structure refinement for **AgNaInSbHoErBi**.

Compound name	**AgNaInSbHoErBi**
Empirical formula	$Ag_{0.33}Bi_{0.24}Cl_6Cs_2Er_{0.08}Ho_{0.13}In_{0.33}Na_{0.67}Sb_{0.22}$
Formula weight	679.17
Temperature/K	173.15
Crystal system	cubic
Space group	*Fm-3m*
a/Å	10.5864(2)
b/Å	10.5864(2)
c/Å	10.5864(2)
α/°	90
β/°	90
γ/°	90
Volume/Å^3	1186.44(7)
Z	4
ρ_{calc}g/cm^3	3.802
μ/mm^{-1}	7.420
F(000)	1185.0

Crystal size/mm^3	0.05 × 0.05 × 0.05
Radiation	AgKα (λ = 0.56086)
2Θ range for data collection/°	5.26 to 40.658
Index ranges	-13 ≤ h ≤ 13, -13 ≤ k ≤ 11, -13 ≤ l ≤ 13
Reflections collected	3784
Independent reflections	87 [R_{int} = 0.0380, R_{sigma} = 0.0146]
Data/restraints/parameters	87/0/8
Goodness-of-fit on F^2	1.166
Final R indexes [I>=2σ (I)]	R_1 = 0.0150, wR_2 = 0.0382
Final R indexes [all data]	R_1 = 0.0150, wR_2 = 0.0382
Largest diff. peak/hole / e Å^{-3}	1.39/-0.79

Table S3. Fractional Atomic Coordinates (×10^4) and Equivalent Isotropic Displacement Parameters (Å^2×10^3) for **AgNaInSbHoErBi**. U_{eq} is defined as 1/3 of the trace of the orthogonalised U_{IJ} tensor.

**Atom**	*x*	*y*	*z*	**U(eq)**
Cs1	7500	7500	2500	17.9(3)
Cl1	5000	5000	2578.1(9)	20.4(3)
Ag1	5000	5000	0	9.9(6)
In1	5000	5000	5000	8.0(3)
Na1A	5000	5000	0	9.9(6)
Er1	5000	5000	5000	8.0(3)
Sb1	5000	5000	5000	8.0(3)
Bi1	5000	5000	5000	8.0(3)
Ho1	5000	5000	5000	8.0(3)

Table S4. Anisotropic Displacement Parameters (Å^2×10^3) for **AgNaInSbHoErBi**. The Anisotropic displacement factor exponent takes the form: -$2\pi^2[h^2a^{*2}U_{11}+2hka^*b^*U_{12}+...]$.

**Atom**	U_{11}	U_{22}	U_{33}	U_{23}	U_{13}	U_{12}
Cs1	17.9(3)	17.9(3)	17.9(3)	0	0	0
Cl1	24.4(4)	24.4(4)	12.4(4)	0	0	0
Ag1	9.9(6)	9.9(6)	9.9(6)	0	0	0
Na1A	9.9(6)	9.9(6)	9.9(6)	0	0	0

Table S5. The PL quantum yield for HE-DPs.

	AgNaInSbHoErBi	InSbHoErYb	InSbHoErBi	InSbHoErDy	InSbHoErTb
λ_{em}=400 ~ 800nm	0.3%	2.4%	1.1%	1.9%	1.8%
λ_{em}=1000 ~ 1700nm	8.7%	2.6%	3.8%	0.8%	8.5%

Table S6. TRPL results for HE-DPs and $Cs_2NaInCl_6$:Sb.

	AgNaInSbHoErBi	InSbHoErYb	InSbHoErBi	InSbHoErDy	InSbHoErTb	$Cs_2NaInCl_6$:Sb
λ_{em}=440 nm / μs	0.241	0.543	0.332	0.460	0.696	0.651
λ_{em}=550 nm / ms	1.97	0.892	1.40	1.23	1.66	n/a
λ_{em}=488 nm / ms	2.05	1.57	1.17	1.34	2.06	n/a
λ_{em}=655 nm / ms	7.83	1.64	1.84	0.96	1.69	n/a
